# Revision Laparoscopic Adjustable Gastric Band as a Successful Alternative Over Sleeve Gastrectomy After Failed Initial Weight Loss Therapies

**DOI:** 10.7759/cureus.14969

**Published:** 2021-05-11

**Authors:** Enkhmaa Luvsannyam, Sivasthikka Lingarajah, Molly S Jain, Kajal Goraya, Bernard O Emuze, Jay Sanni, Frederick Tiesenga

**Affiliations:** 1 Research, California Institute of Behavioral Neurosciences & Psychology, Fairfield, USA; 2 Research and Development, Windsor University School of Medicine, Frankfort, USA; 3 Medicine, Saint James School of Medicine, Park Ridge, USA; 4 Medicine, Caribbean Medical University, Willemsted, CUW; 5 Emergency Medicine, Saint James School of Medicine, Park Ridge, USA; 6 Family Medicine, Windsor University School of Medicine, Frankfort, USA; 7 General Surgery, West Suburban Medical Center, Chicago, USA

**Keywords:** morbid obesity, bariatric surgery, laparoscopic adjustable gastric band, sleeve gastrectomy, weight loss

## Abstract

Bariatric surgery is one of the most successful treatment options for morbid obesity and related comorbidities that is reserved for patients when lifestyle modifications and medical treatments fail. Bariatric surgeries are proven to result in weight reduction and improve obesity-related complications; however, there still are some reported failures. We report the case of a 35-year-old woman with morbid obesity and diabetes mellitus who had failed laparoscopic adjustable gastric band (LAGB) and laparoscopic sleeve gastrectomy (LSG) when done individually. The patient finally had a successful weight loss after undergoing revision LAGB over LSG. Although the present literature reports LAGB being an unsuccessful weight loss procedure, this case highlights the significance of LAGB as an effective bariatric surgery compared to other procedures. Our patient not only lost her weight successfully but also resolved her comorbid conditions and mental illness following the LAGB.

## Introduction

Obesity is a major health problem in the United States, and the prevalence rates have been increasing over time. The latest research shows that 34% of adults and 15-20% of children and adolescents in the United States are obese [1]. This epidemic has increased the risk of chronic diseases such as diabetes and hypertension in this population. Although various treatment options are available for the management of morbid obesity, bariatric surgeries are proven to be the most successful modality to reduce overall weight and obesity-related comorbidities [2]. The criteria for surgical intervention for weight loss recognized by a National Institutes of Health consensus panel include the failure of medical treatment and patients with body mass index (BMI) of ≥40 kg/m^2^ or 35-40 kg/m^2^ with associated comorbid conditions.

The different types of bariatric surgeries include laparoscopic sleeve gastrectomy (LSG), Roux en-Y gastric bypass (RYGB), and laparoscopic adjustable gastric band (LAGB) [3]. LAGB over LSG is a rare procedure that is not ideally utilized by many physicians; however, a recent study showed a promising result of LAGB as a revision surgery after a failed bariatric procedure [2]. The study showed a group of patients with a mean estimate weight loss of 30.75 lbs and BMI reduction from 46.91 kg/m^2^ to 40.7 kg/m^2^ two years after the LAGB as a revision procedure [2].

In this report, we present a case of successful weight loss of a 35-year-old patient who underwent successful LAGB as a revision surgery following a failed LAGB and LSG individually.

## Case presentation

A 35-year-old female with morbid obesity and type II diabetes mellitus was referred by her primary care physician (PCP) for weight-loss management due to uncontrolled blood sugar levels and inability to lose weight in 2010. The patient’s weight was 325 lbs with a BMI of 53.5 kg/m^2^ and hemoglobin A1c (HbA1c) of 12.3%. She was referred to a dietician and was following a high-protein, low-carbohydrate, and low-fat bariatric diet. The patient was also exercising regularly with the consultation of a personal trainer. Nevertheless, she was not able to reach her goal weight BMI of 30 kg/m^2^. The patient was prescribed phentermine by her PCP for six months to lose weight which was not effective enough. After reviewing the patient’s past medical history and her extensive efforts in losing weight for many years, the patient qualified as an eligible candidate for bariatric surgery. The patient was further provided information on different types of bariatric surgeries as well as the risks and benefits of each type of surgery. The patient was more interested in LAGB due to complete reversibility and underwent the surgery in 2010. One-year post-surgery, her BMI decreased from 53.5 to 31.1 kg/m^2^ and HbA1c reduced to 8.2%.

In 2013, the patient revisited the clinic and complained that she has nausea and constant vomiting up to four times a day. Additionally, she could not keep liquids and solids down for the past four weeks. The patient also experienced constant heartburn and occasional dysphagia. It was evident on a computed tomography scan that port leakage was causing her aforementioned symptoms. She requested if there were other options to replace LAGB. After careful discussion and review, the patient decided to get LAGB removed and replace it with LSG simultaneously. Initially, the patient had no major post-operative complications and responded well with LSG. The patient was engaged in post-surgery support groups which incorporated weight loss plans, including aerobic exercises such as walking, biking, and swimming. She also added yoga and cardio machines in her routine for five days a week. The patient strictly followed an 800-calorie bariatric diet that included vegetables, fruits, whole grains, high protein, low carbohydrate, and low fat. She was also taking daily bariatric multivitamins two times a day.

The patient returned to the clinic in 2014 with a BMI of 40.3 kg/m^2^ and complained that she was unable to lose weight with LSG despite following the required bariatric diet, increased physical activity, and lifestyle modifications. The patient was surprised that she was unable to restrict the size of eating portions as well. This had an effect on her mental health, contributing to psychological issues including depression and anxiety. The patient started consulting a psychiatrist to deal with her mentioned psychiatric disorders. She refused to have any other types of weight loss procedures since then, stating that she only wanted to return back to LAGB that she had lost weight successfully in the past.

**Figure 1:**
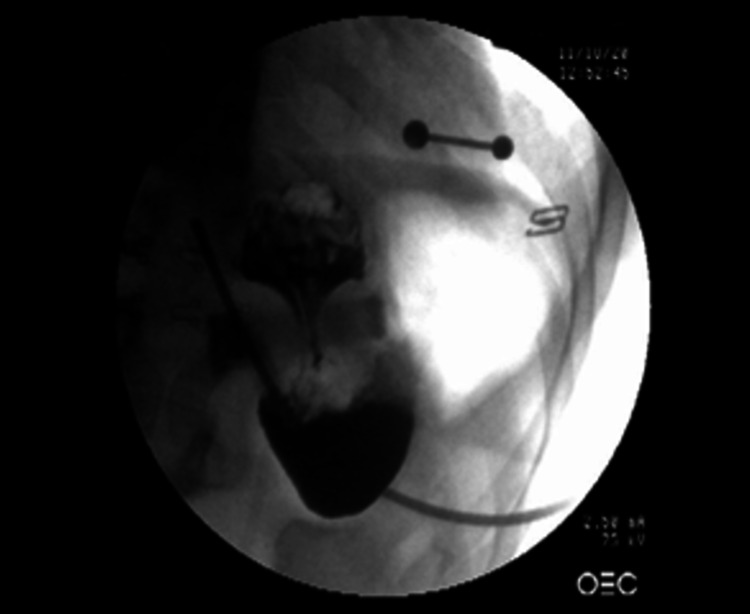
Post-operative fluoroscopic image of the lap band adjustment.

After careful evaluations, as per the patient’s request, LAGB over LSG was performed in 2015 as revision surgery. The patient consistently followed up for weight loss evaluations every four weeks and the lap band was optimally adjusted using fluoroscopy (Figure 1).

The patient was continuing a bariatric diet and multivitamin as per advice from a dietician. She was also participating in the support group and exercising consistently. Presently, six years post-surgery, the patient weighs 189 lbs with a BMI of 31.1 kg/m^2^. The patient is pleased with the outcome that she did not experience any gastrointestinal symptoms and never regained weight post-surgery. Her diabetes is also well controlled with HbA1c of 6.7% after the weight loss, and she successfully recovered from depression and anxiety as well. The patient is satisfied that she significantly benefited from undergoing revision surgery of LAGB over the failed LSG procedure.

## Discussion

Bariatric surgical interventions have significantly contributed to a major breakthrough in the management of obesity in the United States. Some of the more complicated procedures such as RYGB and biliopancreatic diversion with duodenal switch have the highest success rate of weight loss. However, they are associated with higher morbidity and mortality rates [2]. Presently, LAGB and LSG are among the major bariatric surgeries that are gaining recognition. Both the procedures are associated with significant advantages of higher achievable weight loss, lesser operative times, reduced mineral or electrolyte deficiencies, lower rate of complications, and efficacy at reducing comorbid conditions related to obesity [4].

The LAGB method is known for its higher flexibility and minimal invasiveness [2]. The adjustable gastric band is advantageous for patients to get it adjusted accordingly with each follow-up to their comfort of maintaining the lifestyle modifications of eating habits. However, this flexibility could also lead to significant weight gain when patients fail to comply with their dietary modifications. Additionally, less common complications such as band slippage, pouch dilatation, ineffective esophageal motility, and hiatal hernia are possibilities that lead to revision surgical procedures in correction to LAGB failure [5]. LSG is another bariatric surgery that is more invasive than LAGB but has a better weight loss rate [3]. This procedure involves resection of 80% of the stomach, creating a tubular structure involving lesser curvature of the stomach [2]. Some known complications with this procedure include post-operative leakage, bleeding, and dumping syndrome [3]. Moreover, once patients start consuming larger portions of their diet, there are chances of regaining significant weight and subsequent procedure failure.

The majority of studies suggest that presently, LAGB is not the most effective surgical management for weight loss [4-7]. Observational studies found that post-operative complications increased with LAGB procedure along with increasing chances of failure rate as a growing concern [4,5]. The higher rates of reoperation and decreased efficacy with LAGB have led bariatric surgeons to compare LAGB even with LSG or RYGB, which are much better in their goals of achieving weight loss and avoiding readmission [4]. A study by O’Brien et al. demonstrated the revision rate for the failure of LAGB to about 40% in the first 10 years. The study also elaborated on LAGB patients suffering complications such as port and tubing issues, erosion formation, and proximal enlargement requiring further intervention [6]. Another systematic review by Egberts et al. reinforced the proximal pouch enlargement as a major complication of LAGB causing a reoperation rate of 5% within two years [7].

The highlight of this case pertains to the success of LAGB, although it has been reported to be one of the most inefficient ways of weight loss management. The patient, in this case, suffered port leakage as a complication of her first LAGB procedure and switched to LSG; however, she regained her weight due to failed LSG. The failure of LSG in her case not only made her comorbid condition of diabetes worse but also led her towards depression. She ultimately made her choice to go back to LAGB but with a second revision found the best aid with this surgery. The patient not only lost her weight to her desired goal but had better control of her diabetes, and her mental health also improved. Moreover, the patient suffered no major intra-operative or post-operative complications with the second revision surgery of LAGB. Although studies support the success of other bariatric surgeries as a better means of weight loss in comparison to LAGB, our case presents a unique representation of LAGB success. Finally, this report supports the fact that LAGB could be a preferable option in “selective” patient populations such as young patients, females planning a pregnancy, older patients, and patients with complicated underlying conditions. LAGB still remains a beneficial option due to decreased operative times, flexibility of reversibility, and for patients who cannot tolerate complex surgeries.

## Conclusions

Bariatric surgery is the last option for the management of morbid obesity. Even though most patients benefit from bariatric surgeries and have successful weight reduction, there are still many patients suffering from a failed treatment despite strict post-surgery follow-ups, diet, and exercise. This case brings attention to careful evaluation and choosing the appropriate surgical option based on individual patient history and preference. It also proves that the LAGB procedure performed well for this patient despite the current literature supporting that LAGB is not as successful as other bariatric surgeries. Obesity management by weight loss surgery should be tailored to the specific needs of each patient. It should not only focus on weight loss but also on the physical, mental, and emotional wellbeing of the patient. Finally, when individual patient needs are addressed, a multidisciplinary method will provide a stronger outcome and quality of life.
